# Simulation of heat transfer in a landfill with layered new and old municipal solid waste

**DOI:** 10.1038/s41598-022-06722-6

**Published:** 2022-02-22

**Authors:** Tao Zhang, Jianyong Shi, Xun Wu, Shi Shu, Hai Lin

**Affiliations:** 1 School of Civil Engineering and Architecture, Nanchang Hangkong University, Nanchang, 330063 China; 2 Geotechnical Engineering Research Institute, Hohai University, Nanjing, 210098 China; 3 Key Laboratory of Ministry of Education for Geomechanics and Embankment Engineering, Hohai University, Nanjing, 210098 China; 4 School of Civil Engineering and Architecture, Nanchang University, Nanchang, 330031 China

**Keywords:** Environmental sciences, Solid Earth sciences

## Abstract

Due to rapid degradation of the newly filled municipal solid waste (MSW), the local temperature of the waste layer increases greatly. The mechanical parameters related to waste degradation and the deformation of high-density polyethylene (HDPE) pipes in the waste body will be affected by the elevated temperature. To predict the temperature distribution in the anaerobic landfill, a one-dimensional heat transfer model is established in this study. This model considers the stratification of the saturated and unsaturated zones, and the layering of new and old waste. Furthermore, a single peak model for heat production is applied as the source term of heat production. The stratification of the unsaturated and saturated zones is considered by distinguishing the difference in heat conductivity and specific heat capacity. The layering of the new and old waste layers is considered by distinguishing the difference in the length of time that waste has been degraded to produce heat. Based on the numerical calculation method, the temperature distribution in a landfill with layered new and old MSW is well simulated. The position where the maximum temperature occurs and the variation in the temperature at the edge of new and old waste are elucidated. The sensitivity analysis shows that the influence of the density on the temperature distribution is more significant. Besides, the stratification of saturated–unsaturated waste should also be considered in landfills.

## Introduction

The biodegradation reaction causes an increase in waste temperature, and the normal working performance of HDPE pipes and the geomembrane in the landfill will be affected by high temperatures^1,2^. In addition, the temperature also affects the mechanical behavior of the waste pile^3^, which increases the uncertainty in the stability analysis of the landfill^4^. Therefore, to ensure the safe operation of landfills, the exploration of the thermal response has become an important research interest. Rees^5^, Spokas and Bogner^6^, Hanson et al.^7–9^, Yesiller and Hanson^10^, Yesiller et al.^11,12^, Bonany^13^, Shariatmadari et al.^14^, Hunte et al.^15^, Koerner and Koerner^16^, and Vaverkova and Adamcova^17^ investigated the waste temperature in different landfills. It was found that a shallow waste temperature was influenced by atmospheric temperature. In terms of the temperature distribution, Spokas and Bogner^6^ and Van Elk^18^ found that waste temperature increased with depth; Yoshida and Rowe^19^, Hanson et al.^9,20^, Reinhart et al.^21^, and Jafari et al.^22,23^ indicated that the maximum temperature was near the middle of the landfill; Zhang et al.^24^ analyzed the temperature distribution in a newly filled waste layer and concluded that the highest temperature appeared near the leachate level. In terms of the variation in temperature over time, Lefebvre et al.^25^, Hanson et al.^7^, Bouazza et al.^26^, Jafari et al.^22^, and Nocko et al.^27^ pointed out that the waste temperature increased rapidly in the early stage. Yoshida and Rowe^19^ found that the waste temperature began to decrease approximately 10 years later. Magyar and Faitli^28^ summarized the temperature variations of different filling age wastes in the Gyal landfill, and proposed a trend function for the temperature variation; Moreau et al.^29^ found that the waste temperature in the Caen landfill increased significantly during the waste placement period, but the waste temperature decreased after the closure of the landfill. The stratification of the new and old waste existed in an operating landfill^24,25,30,31^. The temperature variations in this kind of landfill also should be estimated using theoretical calculations.

Currently, many scholars have obtained the temperature distribution in landfills using numerical^19,29,32–44^ and analytical^45,46^ calculations. The landfills in these studies were generally regarded as landfills with a homogeneously unsaturated waste layer^19,29,32–46^ or a continuously placed waste layer^19,32,33,35,36,39–43,45,46^. The current theoretical method can be used to evaluate the temperature distribution in most landfills. However, the current theoretical methods used to predict the variation trend and longitudinal distribution of temperatures in operating landfills in areas with abundant rainfall may produce significant errors. If it is necessary to predict the temperature in such landfills, the stratification of the new and old waste and the stratification of the saturated–unsaturated waste should be considered in the establishment of theoretical methods.

Based on numerical methods, a one-dimensional heat transfer model for estimating the temperature in a landfill with layered new and old waste and layered saturated and unsaturated waste is presented in this study. The differences in the length of time that waste has been degraded, the specific heat capacity and the heat conductivity in the waste layers are considered in this model. The stratification of the unsaturated and saturated zones is considered by distinguishing the specific heat capacity and heat conductivity of waste in the different zones. The stratification of the new and old waste layers is considered by distinguishing the length of time that the waste has been degraded to produce heat in the different waste layers. The results of the numerical calculation are then compared with the temperature in the newly filled waste layer measured by Zhang et al.^24^. The validity of the theoretical model in simulating the temperature distribution in the newly filled waste layer is determined. The evolution of the temperature distribution in the landfill with the layered new and old waste layers is analyzed. The position where the maximum temperature occurs and the variation in temperature at the edge of the new and old waste are elucidated. In addition, the significant sensitive parameters of the temperature distribution are discovered.

## Materials and methods

### Numerical model of the heat transfer

To establish a model for predicting temperature in landfills, it was assumed that the density, heat conductivity and specific heat capacity of waste are isotropic, and these parameters do not vary with time. According to the law of energy conservation, the heat flowing into the unit body plus the heat brought into the unit body by the fluid plus the heat produced by the unit body are equal to the increase in the internal energy of the unit body, which can be represented using the following equation:

1 $$\lambda \left[ {\frac{{\partial^{2} T}}{{\partial x^{2} }} + \frac{{\partial^{2} T}}{{\partial y^{2} }} + \frac{{\partial^{2} T}}{{\partial z^{2} }}} \right] - \rho_{w} C_{w} \left[ {\frac{{\partial \left( {u_{w} T} \right)}}{\partial x} + \frac{{\partial \left( {v_{w} T} \right)}}{\partial y} + \frac{{\partial \left( {w_{w} T} \right)}}{\partial z}} \right] + Q_{T} = \frac{\partial T}{{\partial t}}\rho C$$

where *λ* is the heat conductivity of waste (W (m K)^-1^); *T* is the temperature in the unit body (℃); *t* is the time; *ρ*_*w*_ is the density of the fluid (kg m^-3^); *u*_*w*_, *v*_*w*_, and *w*_*w*_ are the velocity of the fluid in the direction *x*, *y*, and *z*, respectively (m s^-1^); *ρ* is the density of waste (kg m^-3^); *C* and *C*_*w*_ are the specific heat capacity of the waste and fluid, respectively (J (kg K)^-1^); *Q*_*T*_ is the amount of heat produced by biodegradation per unit volume of waste per unit time (J (m^3^ d)^-1^).

The waste can be divided into easily degraded, moderately degraded, and difficultly degraded according to the biodegradability of the waste^47–49^. According to the equation of waste biodegradation rate presented by Liu et al.^50^, the amount of heat generated by biodegradation per unit volume of waste per unit time can be presented, as shown in the following equation:

2 $$Q_{T} = \sum\limits_{i = 1}^{3} {\omega_{i} \frac{{A_{Ti} }}{{B_{Ti} }}\left( {t + D_{Ti} } \right)e^{{ - \frac{{\left( {t + D_{Ti} } \right)}}{{B_{Ti} }}}} }$$

where *i* = 1, *i* = 2, and *i* = 3 represent easily degraded, moderately degraded, and difficultly degraded components in the waste, respectively; *ω*_*i*_ is the proportion of a component; $A_{T} = {{\rho_{0} q_{T} A} \mathord{\left/ {\vphantom {{\rho_{0} q_{T} A} {M_{w} }}} \right. \kern-\nulldelimiterspace} {M_{w} }}$, and *A*_*Ti*_ are the parameters related to the peak heat production rate (J m^-3^ d^-1^); *ρ*_0_ is the initial density of the waste (kg m^-3^); *q*_*T*_ is the amount of heat generated by the biodegradable per unit amount of substance of waste (J mol^-1^); *A* is the parameter related to the biodegradation (d); *M*_*w*_ is the molar mass of the waste (g mol^-1^); *B*_*Ti*_ is the time of the peak heat production rate (d); The *D*_*Ti*_ is the length of time that the waste has been degraded to produce heat (d).

Substituting Eq. (2) into Eq. (1). The estimation of the temperature distribution in the landfill is a one-dimensional problem, because the heat is only considered to transfer to the outside and into the waste layer. Therefore, the following equation can be obtained:

3 $$\lambda \frac{{\partial^{2} T}}{{\partial z^{2} }} + \sum\limits_{i = 1}^{3} {\omega_{i} \frac{{A_{Ti} }}{{B_{Ti} }}\left( {t + D_{Ti} } \right)e^{{ - \frac{{t + D_{Ti} }}{{B_{Ti} }}}} } = \frac{\partial T}{{\partial t}}\rho C$$

Equation (3) is the one-dimensional transient basic difference equation of the heat transport in the MSW layer. The following equation can be obtained by the difference in Eq. (3):

4 $$f_{T1} \frac{{T_{k - 1}^{t} - 2T_{k}^{t} + T_{k + 1}^{t} }}{{h_{z}^{2} }} + f_{T2} = f_{T3} \frac{{T_{k}^{t} - T_{k}^{t - 1} }}{\tau }$$

where $f_{T1} = \lambda$; $f_{T2} = \sum\nolimits_{i = 1}^{3} {\omega_{i} \frac{{A_{Ti} \left( {t + D_{Ti} } \right)}}{{B_{Ti} }}e^{{ - \frac{{\left( {t + D_{i} } \right)}}{{B_{Ti} }}}} }$; $f_{T3} = \rho C$; *h*_*z*_ is the step length in the vertical direction; *τ* is the step length of time.

Equation (4) is the difference equation of a one-dimensional transient difference scheme for heat transfer in a landfill. The difference equation is in the classical implicit format, indicating that the difference equation can be solved via iterative methods.

## Results and discussion

### Temperature distribution in the Wuxi landfill

**Figure 1:**
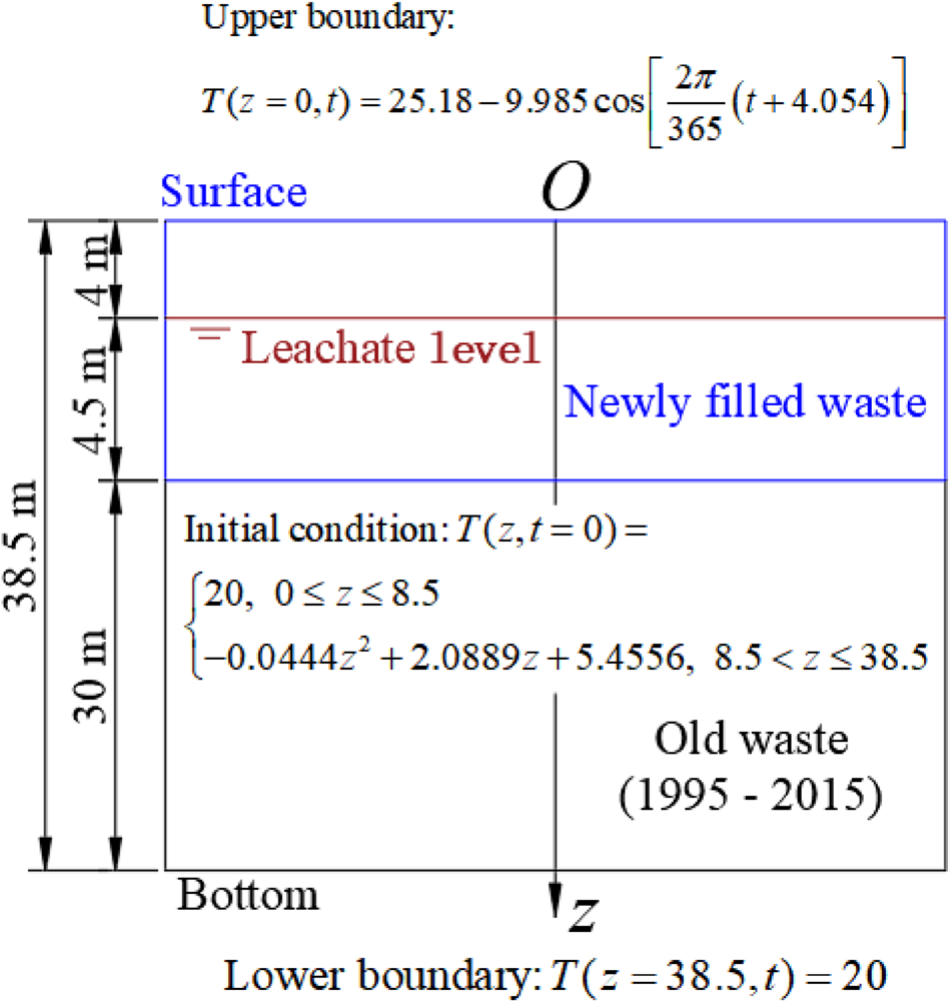
Physical model for heat transfer in the Wuxi landfill.

Wuxi landfill, where Zhang et al.^24^ conducted their field test, is located in the southeast of China. The average annual rainfall is approximately 1900 mm, and the atmospheric temperature ranges from −9 °C to 39°C^24^. Wuxi landfill is a typical valley-type landfill and also an anaerobic landfill. Designed to accommodate 4.20 million m^3^ of MSW. During the year 2016, 2200 t d^−1^ of MSW were placed in this landfill. Zhang et al.^24^ monitored the temperature in the newly filled waste layer from January 18, 2016 to June 18, 2017. The waste at the bottom of the Wuxi landfill has been placed for 20 years. Hanson et al.^9^ observed that the temperature for the waste with a filling age of approximately 20 years was stable at 20 ℃. Therefore, the lower boundary was considered as a constant boundary (the Dirichlet boundary condition) during the process of calculation. The surface temperature of the landfill fluctuates with the atmospheric temperature, and the upper boundary can be represented by a cosine function^36,45,51^. Therefore, the cosine function was used to fit the observed data of the surface temperatures. The fitting results are shown in Fig. 1, and the upper boundary was also the Dirichlet boundary condition. The average initial waste temperature in the newly filled waste layer was found to be approximately 20 ℃. However, the temperature of the measuring point was still the measured temperature. Hanson et al.^9^ observed that the temperature of waste with a filling age of approximately 10 years in the middle of the landfill was 30 ℃, and it was also found that the temperature in the middle of the landfill was the highest. Then the quadratic function was used to simulate the temperature distribution in the old waste layer. The physical model for simulating heat transfer in the Wuxi landfill is shown in Fig. 1.

According to Eq. (3) and Fig. 1, the following mathematical model was obtained:

5 $$\left\{ \begin{gathered} {\text{Governing}}\;{\text{equation}}:\;\lambda \frac{{\partial^{2} T}}{{\partial z^{2} }} + \sum\limits_{i = 1}^{3} {\omega_{i} \frac{{A_{Ti} }}{{B_{Ti} }}\left( {t + D_{Ti} } \right)e^{{ - \frac{{t + D_{Ti} }}{{B_{Ti} }}}} } = \frac{\partial T}{{\partial t}}\rho C \hfill \\ {\text{Initial}}\;{\text{condition:}}\;T(z,t = 0) = \left\{ \begin{gathered} 20,\;\;0 \le z \le 8.5 \hfill \\ - 0.0444z^{2} + 2.0889z + 5.4556,\;\;8.5 < z \le 38.5 \hfill \\ \end{gathered} \right. \hfill \\ {\text{Upper}}\;{\text{condition:}}\;T(z = 0,t) = 25.18 - 9.985\cos \left[ {\frac{2\pi }{{365}}\left( {t + 4.054} \right)} \right] \hfill \\ {\text{Lower}}\;{\text{condition:}}\;T(z = 38.5,t) = 20 \hfill \\ \end{gathered} \right.$$

When Eq. (5) is differenced and according to Eq. (4), the following mathematical model of difference scheme was obtained:

6 $$\left\{ \begin{gathered} {\text{Governing}}\;{\text{equation}}:\;f_{T1} \frac{{T_{k - 1}^{t} - 2T_{k}^{t} + T_{k + 1}^{t} }}{{h_{z}^{2} }} + f_{T2} = f_{T3} \frac{{T_{k}^{t} - T_{k}^{t - 1} }}{\tau } \hfill \\ {\text{Initial}}\;{\text{condition:}}\;T_{k}^{t = ot(0)} = \left\{ \begin{gathered} 20,\;\;0 \le \left[ {oz(z) - 1} \right]h_{z} \le 8.5 \hfill \\ - 0.0444\left\{ {\left[ {oz(z) - 1} \right]h_{z} } \right\}^{2} + 2.0889\left[ {oz(z) - 1} \right]h_{z} \hfill \\ + 5.4556,\;\;8.5 < \left[ {oz(z) - 1} \right]h_{z} \le 38.5 \hfill \\ \end{gathered} \right. \hfill \\ {\text{Upper}}\;{\text{condition:}}\;T_{z = oz(0)}^{t} = 25.18 - 9.985\cos \left\{ {\frac{2\pi }{{365}}\left[ {\left( {ot(t) - 1} \right)\tau + 4.054} \right]} \right\} \hfill \\ {\text{Lower}}\;{\text{condition:}}\;T_{z = oz(38.5)}^{t} = 20 \hfill \\ \end{gathered} \right.$$

where *ot*(*t*) and *oz*(*z*) are the node functions in time and the vertical direction, respectively.

**Figure 2:**
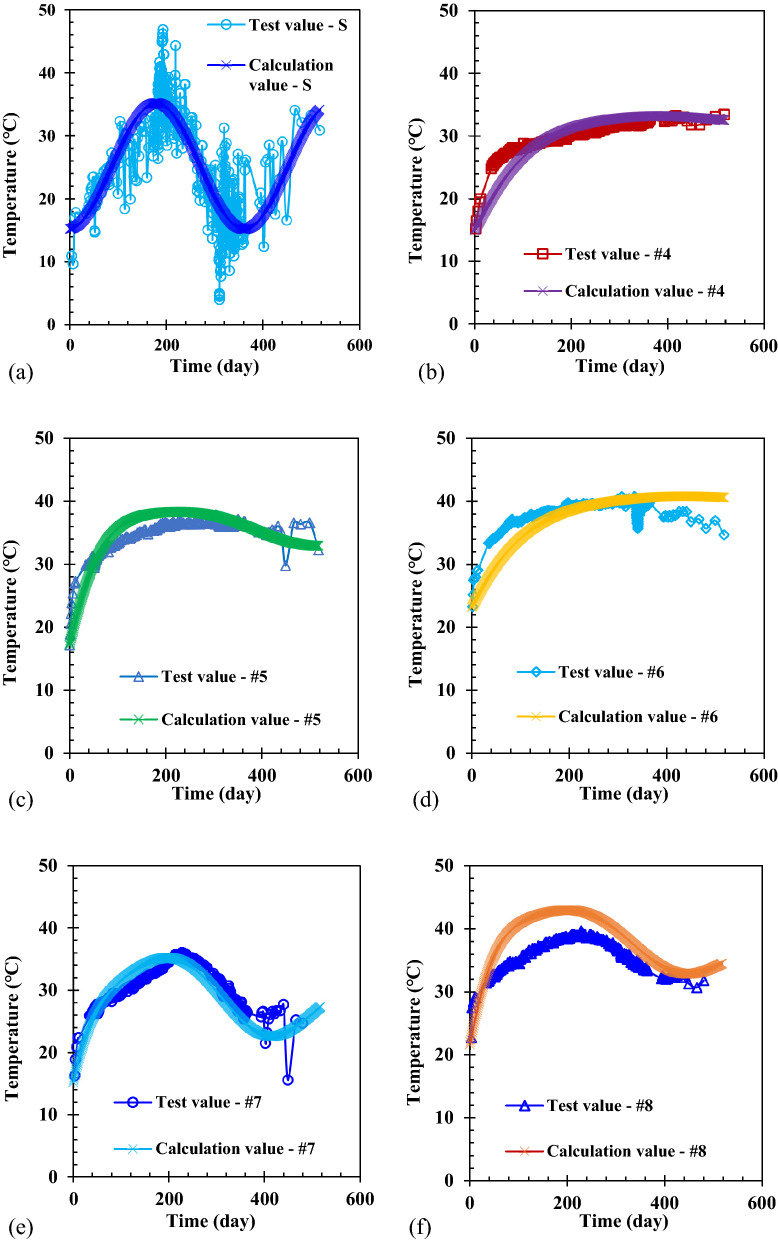
Comparison of the calculation and test values of temperature with time at the bottom of the different wells: (**a**) Surface (Depth: 0 m); (**b**) #4 well (Depth: 5.88 m); (**c**) #5 well (Depth: 3.58 m); (**d**) #6 well (Depth: 6.56 m); (**e**) #7 well (Depth: 1.34 m); (f) #8 well (Depth: 2.05 m).

In order to select reasonable calculation parameters, the parameters collected from the Wuxi landfill and those suitable for the Wuxi landfill were selected as far as possible. The parameters used in the numerical calculation are shown in Table 1. The variation in the waste temperature with time in the newly filled MSW layer of the Wuxi landfill was obtained using numerical calculation, as shown in Fig. 2. Although the local surface temperature fluctuated greatly due to the random change in weather, the surface temperature was higher during the summer and lower during the winter in general. Therefore, this rule was well expressed using the surface temperature curve fitted by a cosine function in this study. In the earlier period after the waste was placed in the landfill, the waste biodegradation was relatively rapid, and more cellulose and hemicellulose were involved in the biodegradation reaction^50^. Then more heat was produced by the aerobic and anaerobic reactions of the waste. Therefore, the waste temperature increased faster during this period, and vice versa during the later period. In addition, the waste temperature in the shallow layer was easily influenced by the atmospheric fluctuation after the waste temperature reached the peak temperature. This was due to the reduced rate of biodegradation and the heat production from non-dominant biodegradation. The calculation value is compared with the field test value in Fig. 2, and it can be also seen that the variation in temperature in the newly filled MSW layer with time was better simulated using the calculation method and the numerical model presented in this study.

**Table 1:** Parameters of heat transfer model in the Wuxi landfill.

Parameter	Value	Value from reference	Reference
*λ* (W (m K)^-1^)	$$\left\{ \begin{gathered} 0.1,\;{\text{Unsaturated}}\;{\text{zone}} \hfill \\ 0.2,\;{\text{Saturated}}\;{\text{zone}} \hfill \\ \end{gathered} \right.^{{\text{a}}}$$	0.035—0.242	^52^
*C* (J (kg K)^-1^)	$$\left\{ \begin{gathered} 557.4,\;{\text{Unsaturated}}\;{\text{zone}} \hfill \\ 1376.2,\;{\text{Saturated}}\;{\text{zone}} \hfill \\ \end{gathered} \right.^{{\text{b}}}$$	–	^53,54^
*ρ* (kg m^-3^)	700.0	–	^24^
*D*_*G*_ (d)	$$D_{T} = \left\{ {\begin{array}{*{20}c} {0,\;{\text{Newly}}\;{\text{filled}}\;{\text{waste}}\;{\text{layer}}}^{{\text{c}}} \\ {40 + \frac{z - 8.5}{{1.5}} \times 365,\;{\text{Old waste layer}}} \\ \end{array} } \right.$$	–	–
*ω*_1_, *ω*_2_ and *ω*_3_	0.15, 0.55 and 0.30	0.15, 0.55 and 0.30	^47–49^
*A*_*T*1_, *A*_*T*2_ and *A*_*T*3_ (W m^-3^)	15.0, 5.0 and 0.6^d^	–	^9,24,55^
*B*_*T*1_, *B*_*T*2_ and *B*_*T*3_ (d)	30, 50 and 350^d^	–	^9,24,55^

^a^The heat conductivity of waste in the saturated zone was larger than that in the unsaturated zone^19,33^, and then the heat conductivities of waste in the saturated zone and the unsaturated zone were reasonably selected within the range of the literature value;

^b^Based on the equation of the specific heat capacity of waste provided by Garg and Achari^35^, as shown in Eq. (7), the specific heat capacities for the unsaturated and saturated zones can be calculated according to the waste composition of Wuxi landfill provided by Zhang et al.^24^, and the specific heat capacity of each component provided by Yoshida et al.^53^ and Miller and Clesceri^54^.

$$C = \frac{{C_{s} \left( {1 - n} \right)\rho_{s} + C_{l} nS_{l} \rho_{l} + C_{g} n\left( {1 - S_{l} } \right)\rho_{g} }}{\rho }\qquad (7)$$

where, *C* is the specific heat capacity of waste (J (kg K)^-1^); *C*_*s*_, *C*_*l*_ and *C*_*g*_ are the specific heat capacities of each component, leachate, and landfill gas, respectively (J (kg K)^-1^); *ρ*, *ρ*_*s*_, *ρ*_*l*_ and *ρ*_*g*_ are the densities of waste, each component, leachate, and landfill gas, respectively (kg m^-3^); *n* is the porosity of the waste; *S*_*l*_ is the liquid phase saturation.

^c^In order to simulate the temperature at the bottom of monitoring well, which was placed after the waste body was excavated, the length of time that the waste in the new waste layer had been degraded to produce the temperature was set to 0; 40 was the average length of time for the existence of the new waste layer (d); 8.5 was the thickness of the new waste layer (m); 1.5 was the average height for placing the waste in the old waste layer each year (m);

**Figure 3:**
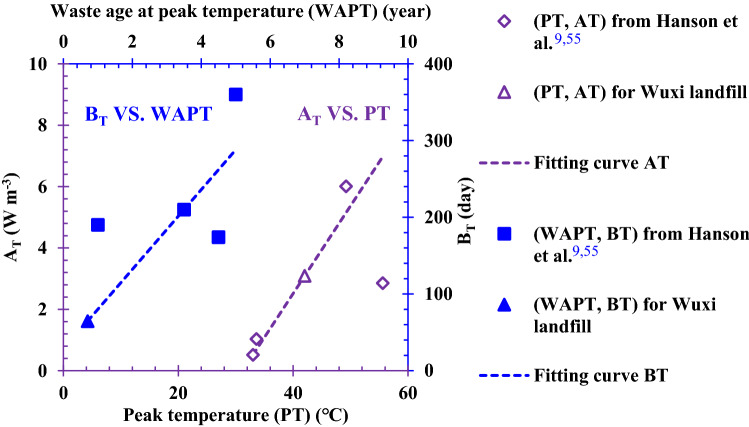
Method of empirical fitting for *A*_*T*_ and *B*_*T*_.

^d^*A*_*T*_/*e* was the peak value of heat production rate in this model, so *A*_*T*_ in Fig. 3 was equal to the peak heat rate in the literature multiplied by *e* (natural constant). $A_{T} = \omega_{i} \sum\nolimits_{i = 1}^{3} {A_{{T_{i} }} }$ and $B_{T} = \omega_{i} \sum\nolimits_{i = 1}^{3} {B_{{T_{i} }} }$ were based on the data provided by Hanson et al.^9,55^ and the actual situation of the Wuxi landfill, *A*_*T*i_ and *B*_*T*i_ were obtained using the method of empirical fitting, as shown in Fig. 3.

**Figure 4:**
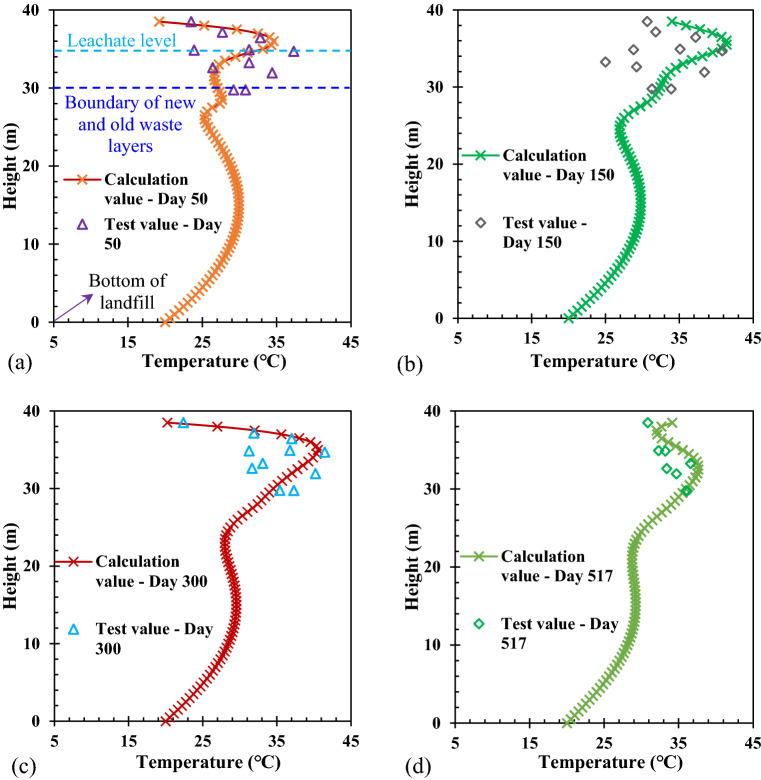
Comparison of the calculation and test values of the temperature with the height at the different times: (**a**) Day 50; (**b**) Day 150; (**c**) Day 300; (**d**) Day 517.

The variations in the waste temperature with height in the Wuxi landfill are shown in Fig. 4. The waste in the old MSW layer had been degraded for many years, the waste biodegradation was relatively slow, and less cellulose and hemicellulose were involved in the biodegradation reaction. Then less heat was produced by the own degradation of the old waste. Therefore, the waste temperature in the old waste layer varied rarely, which was mainly affected by the heat conduction of the waste in the new waste layer. In addition, the closer to the new waste layer, the greater the influence on the temperature of the old waste. In the newly filled waste layer, the waste biodegradation was relatively fast, and more cellulose and hemicellulose were involved in the biodegradation reaction. Then more heat was produced by the own degradation of the new waste, which led to an increase in the new waste temperature. However, the increasing rate of temperature in the middle of the newly filled waste layer was faster than that at both ends. This was because a portion of the heat generated by the waste near the surface and the boundary between the old and new waste was transferred to the atmosphere and the old waste layer in the form of heat conduction, respectively. Another reason why the highest waste temperature occurred near the leachate level was that the specific heat capacity of the waste in the unsaturated zone was less than that in the saturated zone^19^. If the same amount of heat was generated by biodegradation, the increment in temperature in the saturated waste was smaller than that of the unsaturated waste. Then the waste temperature below the leachate level did not increase with depth. When the biodegradation rate in the newly filled waste layer decreased, the increasing rate of the temperature also slowed down until the waste temperature increased to the peak value. After that, the heat was transferred by heat conduction from the site with a higher temperature to the site with a lower temperature until a new heat equilibrium was reached. It can be also seen from Fig. 4 that the calculation method and the numerical model used in this study better reflected the temperature distribution in the new and old waste layers by comparing the calculation value with the test value.

### Sensitivity analysis

**Figure 5:**
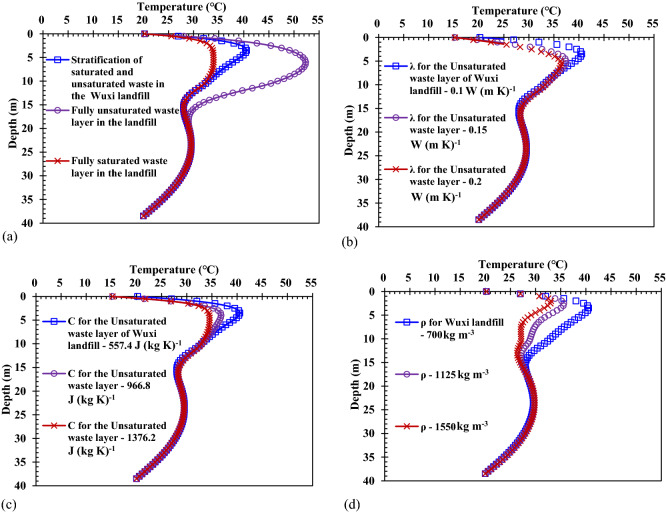
Comparison of the temperature distribution on Day 365 under the conditions of the different sensitive parameters: (**a**) stratification of the saturated–unsaturated waste; (**b**) heat conductivity of the waste (*λ*); (**c**) specific heat capacity of the waste (*C*); (**d**) density of the waste (*ρ*).

A comparison of the temperature distribution under the conditions of the different division methods for the saturated–unsaturated waste zones is shown in Fig. 5a. If the entire waste layer in the landfills was regarded as a fully unsaturated zone, the waste temperature in a new waste layer would be greater than that under the condition of stratification of the saturated–unsaturated waste. Because the saturated zone in the saturated–unsaturated zones was considered as an unsaturated zone, the heat conductivity and specific heat capacity of the waste in the saturated zone needed to take the corresponding values in the unsaturated zone. The heat conductivity of the waste in the unsaturated zone was smaller than that in the saturated zone. When the same amount of heat was generated due to the waste biodegradation, the temperature of the equivalent mass of waste in the fully unsaturated zone increased significantly. Moreover, the heat conductivity of the waste in the fully unsaturated zone was relatively small, then the heat transfer to the surrounding area was relatively slow. Therefore, when the entire waste layer in landfills were regarded as a fully unsaturated zone, the estimated value of the temperature in the new waste layer will be larger, and vice versa.

A comparison of the temperature distribution under the conditions of the different heat conductivities of the waste in the unsaturated waste zone is shown in Fig. 5b. The smaller the heat conductivity of waste in the unsaturated waste zone, the higher the waste temperature in the newly filled MSW layer. This was because if the heat conductivity of waste in the unsaturated zone was relatively small, the heat generated by the waste biodegradation have difficulty transferring to the surrounding area, hence, the heat would accumulate in the original position. In addition, more heat would be generated by the biodegradation of the new filled waste, which would make the waste temperature increase significantly. A comparison of the temperature distribution under the conditions of the different specific heat capacities of the waste in the unsaturated waste zone is shown in Fig. 5c. The smaller the specific heat capacity of the waste in the unsaturated waste zone, the higher the waste temperature in the newly filled MSW layer. This was because if the specific heat capacity of the waste in the unsaturated zone was relatively small, the same amount of heat generated by the waste biodegradation would make the temperature increment of the equivalent mass of waste increase. A comparison of the temperature distribution under the conditions of the different densities is shown in Fig. 5d. The smaller the density of the waste, the higher the waste temperature in the newly filled MSW layer. This was because when the same amount of heat was generated by the waste biodegradation, if the density of the waste was relatively small, the temperature increment for the same volume of waste was greater under the condition that the specific heat capacity of the waste changed slightly. Due to the small heat production of the old waste in the deeper zone, the temperature difference was not obvious.

Using the mathematical model of heat transfer developed in this study, the primary sensitive parameters to the quantification of temperature were analyzed. If the heat conductivity and specific heat capacity of the waste were relatively small, or the waste layer in the landfill was fully unsaturated zone, then the waste temperature would be relatively high. High temperature was beneficial for the collection of landfill gas. However, the larger temperature has an effect on the deformation of the HDPE pipe in the landfill, and when the temperature exceeds a certain range, it was not conducive to waste biodegradation and gas production from waste. When the heat conductivity, specific heat capacity and density of the waste both increased by 1%, the maximum temperatures decrease by 0.30%, 0.17% and 0.22%, respectively. Therefore, the temperature was most sensitive to the density. It is very important to select parameters that are suitable for a specific landfill when the temperature distribution is estimated. In particular, the heat conductivity and the stratification of the saturated–unsaturated waste layers are irrational, which will cause a large error in the temperature estimation. Furthermore, if it is assumed that the heat conductivity, specific heat capacity and density of the waste increase with time, it can be judged from Fig. 5 that the temperature distribution in the newer waste layer is underestimated, and vice versa.

## Conclusions

A one-dimensional heat transfer model for heat response in a landfill with layered new and old waste was established in this study. This model considered the stratification of the saturated and unsaturated zones, and the layering of new and old waste. Furthermore, a single peak model for heat production was applied as the source term of heat production. The stratification of the unsaturated and saturated zones was considered by distinguishing the difference in heat conductivity and specific heat capacity. The layering of the new and old waste layers was considered by distinguishing the difference in the length of time that waste has been degraded to produce heat. Therefore, the established heat transfer model is suitable for anaerobic layered MSW landfills with leachate level.

Based on the numerical calculation method, the temperature distribution in the new and old waste layers was obtained. After the results of calculation were compared with the test result, the following conclusions were found: (1) The temperature distribution in the landfill with the layered new and old waste was better simulated using the heat transfer model and the calculation method developed in this study; (2) The highest temperature occurred near the leachate level in the newly filled waste layer; (3) The temperature at the boundary of the new and old waste was lower than that in the middle of the new and old waste layers at the earlier period after the new waste was placed; (4) The density was a more sensitive parameter that influenced the temperature distribution; (5) When the temperature distribution needs to be estimated, the stratification of the saturated–unsaturated waste should also be considered. In this study, the heat transfer model, the calculation method, and the selection method of the parameters provide a theoretical basis for evaluating the variation in temperature in an anaerobic landfill with layered new and old waste.

## References

[1] Rowe RK, Islam MZ (2009). Impact of landfill liner time – Temperature history on the service life of HDPE geomembranes. Waste Manag..

[2] Krushelnitzky RP, Brachman RWI (2013). Buried high-density polyethylene pipe deflections at elevated temperatures. Geotext. Geomembr..

[3] Falamaki A, Ghareh S, Homaee M, Alireza HS, Sajjad A, Sattar K, Najmeh M, Majid R, Mehran TM, Mostafa D, Ali N (2020). Laboratory shear strength measurements of municipal solid waste at room and simulated in situ landfill temperature, Barmshoor landfill. Iran. Int. J. Civ. Eng..

[4] Falamaki A, Shafiee A, Shafiee AH (2021). Under and post-construction probabilistic static and seismic slope stability analysis of Barmshour landfill, Shiraz City. Iran. Bull. Eng. Geol. Environ..

[5] Rees JF (1980). Optimisation of methane production and refuse decomposition in landfills by temperature control. J. Chem. Technol. Biotechnol..

[6] Spokas KA, Bogner JE (1996). Field system for continuous measurement of landfill gas pressures and temperatures. Waste Manage. Res..

[7] Hanson, J. L., Yesiller, N. & Kendall, L. A. Integrated temperature and gas analysis at a municipal solid waste landfill. In: Proceedings of 16th International Conference on Soil Mechanics and Geotechnical Engineering: Geotechnology in Harmony with the Global Environment, Osaka, Japan (2005).

[8] Hanson, J., Yeşiller, N., Howard, K. A., Liu, W. L. & Cooper, S. P. Effects of placement conditions on decomposition of municipal solid wastes in cold regions. In: Proceedings of 13th International Conference on Cold Regions Engineering, Maine, USA (2006).

[9] Hanson JL, Yeşiller N, Oettle NK (2010). Spatial and temporal temperature distributions in municipal solid waste landfills. J. Environ. Eng..

[10] Yeşiller, N. & Hanson, J. L. Analysis of temperatures at a municipal solid waste landfill. In: Proceedings Sardinia 2003, Ninth International Waste Management and Landfill Symposium, Cagliari, Italy (2003).

[11] Yesiller N, Hanson JL, Oettle NK, Liu WL (2008). Thermal analysis of cover systems in municipal solid waste landfills. J. Geotech. Geoenviron. Eng..

[12] Yeşiller, N., Hanson, J. L. & Yoshida, H. Landfill temperatures under variable decomposition conditions. In: Geo-Frontiers Congress 2011: Advances in Geotechnical Engineering, Dallas, USA (2011).

[13] Bonany JE, Geel PJV, Gunay HB, Isgor OB (2013). Heat budget for a waste lift placed under freezing conditions at a landfill operated in a northern climate. Waste Manage..

[14] Shariatmadari, N., Mansouri, A. & Zarrabi, M. Monitoring the temperature in a sanitary landfill in Tehran. In: Geo-Frontiers Congress 2011: Advances in Geotechnical Engineering, Dallas, USA (2011).

[15] Hunte, C. A., Hettiaratchi, J. P. A., Meegoda, J. N. & Hettiarachchi, C. H. Performance of a waste cell in cold climate operated as an anaerobic landfill bioreactor. In: Geo-Frontiers Congress 2011, Dallas, USA (2011).

[16] Koerner GR, Koerner RM (2006). Long-term temperature monitoring of geomembranes at dry and wet landfills. Geotext. Geomembr..

[17] Vaverková M, Adamcová D (2015). Long-term temperature monitoring of municipal solid waste landfill. Pol. J. Environ. Stud..

[18] Van Elk A, Mañas LS, Boscov M (2014). Field survey of compressibility of municipal solid waste. Soil Rock..

[19] Yoshida, H. & Rowe, R. K. Consideration of landfill liner temperature. In: Proceedings Sardinia 2003, Ninth International Waste Management and Landfill Symposium, Cagliari, Italy (2003).

[20] Hanson, J. L., Yesiller, N. & Oettle, N. K. Spatial variability of waste temperatures in MSW landfills. In: Proceedings of 2008 Global Waste Management Symposium, Colorado, USA (2008).

[21] Reinhart, D. R., Robert, Mackey, P. E., Levin, S., Joslyn, R. & Motlagh, A. Field investigation of an elevated temperature Florida landfill. In: Geotechnical Frontiers 2017, Orlando, USA (2017).

[22] Jafari NH, Stark TD, Thalhamer T (2017). Progression of Elevated Temperatures in Municipal Solid Waste Landfills. J. Geotech. Geoenviron. Eng..

[23] Jafari NH, Stark TD, Thalhamer T (2017). Spatial and temporal characteristics of elevated temperatures in municipal solid waste landfills. Waste Manag..

[24] Zhang T, Shi JY, Qian XD, Ai YB (2019). Temperature and gas pressure monitoring and leachate pumping tests in a newly filled MSW layer of a landfill. Int. J. Environ. Res..

[25] Lefebvre X, Lanini S, Houi D (2000). The role of aerobic activity on refuse temperature rise, I Landfill experimental study. Waste Manage. Res..

[26] Bouazza A, Nahlawi H, Aylward M (2011). In situ temperature monitoring in an organic-waste landfill cell. J. Geotech. Geoenviron. Eng..

[27] Nocko, L. M., Botelho, K., Morris, J. W. F., Gupta, R. & Mccartney, J. S. Estimate of heat generation rates in MSW landfills based on in-situ temperature monitoring. In: Geotechnical Engineering in the XXI Century: Lessons learned and future challenges, Amsterdam, Netherlands (2019).

[28] Magyar T, Faitli J (2016). Temperature distribution measurements in the Gyál MSW landfill. J. Pol. Miner. Eng. Soc..

[29] Moreau J, Grossin-Debattista L, Mazéas C (2019). Six years temperature monitoring using fibre-optic sensors in a bioreactor landfill. Geosci..

[30] Jang YS, Kim YI (2003). Behavior of a municipal landfill from field measurement data during a waste-disposal period. Environ. Earth Sci..

[31] Zhang T, Shi JY, Qian XD, Ai YB (2019). Temperature monitoring during a water-injection test using a vertical well in a newly filled MSW layer of a landfill. Waste Manage. Res..

[32] Manna L, Zanetti M, Genon G (1999). Modeling biogas production at landfill site. Resour. Conserv. Recycl..

[33] Nastev M, Therrien R, Lefebvre R, Glinas P (2001). Gas production and migration in landfills and geological materials. J. Contam. Hydrol..

[34] Gholamifard S, Eymard R, Duquennoi C (2008). Modeling anaerobic bioreactor landfills in methanogenic phase: long term and short term behaviors. Water Res..

[35] Garg A, Achari G (2010). A comprehensive numerical model simulating gas, heat, and moisture transport in sanitary landfills and methane oxidation in final covers. Environ. Model. Assess..

[36] Liu L, Liang B, Xue Q, Zhao Y, Yang C (2011). The modelling of biochemical-thermal coupling effect on gas generation and transport in MSW landfill. Int. J. Environ. Pollut..

[37] Bonany JE, Geel PJV, Gunay HB, Isgor OB (2013). Simulating waste temperatures in an operating landfill in Québec, Canada. Waste Manage. Res..

[38] Megalla D, Van Geel PJ, Doyle JT (2016). Simulating the heat budget for waste as it is placed within a landfill operating in a northern climate. Waste Manage..

[39] Hanson JL, Yeşiller N, Onnen MT, Liu WL, Oettle NK, Marinos JA (2013). Development of numerical model for predicting heat generation and temperatures in MSW landfills. Waste Manage..

[40] Kutsyi DV (2015). Numerical modeling of landfill gas and heat transport in the deformable MSW landfill body. Part 1: Development of the model. Therm. Eng..

[41] Kumar, G., Kopp, K. B., Reddy, K. R., Hanson, J. L. & Yesiller, N. Incorporating thermal effects in modeling of MSW landfills. In: Proceedings of the 8th International Congress on Environmental Geotechnics: Towards a Sustainable Geoenvironment, Hangzhou, China (2019).

[42] Kumar, G., Reddy, K. R. & McDougall, J. Numerical modeling of coupled biochemical and thermal behavior of municipal solid waste in landfills. Comput. Geotech. 128(2020), 103836 (2020).

[43] Kumar G, Kopp KB, Reddy KR, Hanson JL, Yesiller N (2021). Influence of waste temperatures on long-term landfill performance: Coupled numerical modeling. J. Environ. Eng..

[44] Hao Z, Barlaz MA, Ducoste JJ (2020). Finite-element modeling of landfills to estimate heat generation, transport, and accumulation. J. Geotech. Geoenviron. Eng..

[45] Lei, H., Shi, J. & Wu, X. Temperature variation under the consideration of convection and heat generation in landfills. In: Proceedings of the 8th International Congress on Environmental Geotechnics: Towards a Sustainable Geoenvironment, Hangzhou, China (2019).

[46] Wu X, Shi JY, Lei H, Li YP, Okine L (2019). Analytical solutions of transient heat conduction in multilayered slabs and application to thermal analysis of landfills. J. Cent. S. Univ..

[47] Arigala SG, Tsotsis TT, Webster IA, Yortsos YC, Kattapuram JJ (1995). Gas Generation, Transport, and Extraction in Landfills. J. Environ. Eng..

[48] Yu L, Batlle F, Carrera J, Lloret A (2009). Gas flow to a vertical gas extraction well in deformable MSW landfills. J. Hazard. Mater..

[49] Feng, Q. L., Liu, L., Xue, Q. & Zhao, Y. Landfill gas generation and transport in bioreactor landfill. In: Proceedings of International Symposium on Geoenvironmental Engineering, Hangzhou, China (2010).

[50] Liu XD, Shi JY, Qian XD, Hu YD, Peng GX (2011). One-dimensional model for municipal solid waste (MSW) settlement considering coupled mechanical-hydraulic-gaseous effect and concise calculation. Waste Manage..

[51] Yeşiller N, Hanson JL, Liu WL (2005). Heat generation in municipal solid waste landfills. J. Geotech. Geoenviron. Eng..

[52] Lei, H. Study on the heat generation and heat conduction characteristics of waste in landfills. Master thesis. Hohai University, Nanjing, China (2018) (**In Chinese**).

[53] Yoshida, H., Tanaka, N. & Hozumi, H. Theoretical study on temperature distribution in landfills by three-dimensional heat transport model. In: Proceedings of Sardinia 99, Seventh International Waste Management and Landfill Symposium, Cagliari, Italy (1999).

[54] Miller PA, Clesceri NL (2002). Waste sites as biological reactors: Characterization and modeling. J. Hazard. Mater..

[55] Hanson, J. L., Liu, W. L. & Yesiller, N. Analytical and numerical methodology for modeling temperatures in landfills. In: Proceedings of Selected Sessions of GeoCongress 2008: Geotechnics of Waste Management and Remediation, Louisiana, USA (2008).

